# Multi-Population Genetic Algorithm for Multilabel Feature Selection Based on Label Complementary Communication

**DOI:** 10.3390/e22080876

**Published:** 2020-08-10

**Authors:** Jaegyun Park, Min-Woo Park, Dae-Won Kim, Jaesung Lee

**Affiliations:** School of Computer Science and Engineering, Chung-Ang University, 221, Heukseok-Dong, Dongjak-Gu, Seoul 06974, Korea; jgp0566.cau@gmail.com (J.P.); mwpark1711@gmail.com (M.-W.P.)

**Keywords:** communication, evolutionary algorithm, multilabel feature selection, multi-population genetic algorithm

## Abstract

Multilabel feature selection is an effective preprocessing step for improving multilabel classification accuracy, because it highlights discriminative features for multiple labels. Recently, multi-population genetic algorithms have gained significant attention with regard to feature selection studies. This is owing to their enhanced search capability when compared to that of traditional genetic algorithms that are based on communication among multiple populations. However, conventional methods employ a simple communication process without adapting it to the multilabel feature selection problem, which results in poor-quality final solutions. In this paper, we propose a new multi-population genetic algorithm, based on a novel communication process, which is specialized for the multilabel feature selection problem. Our experimental results on 17 multilabel datasets demonstrate that the proposed method is superior to other multi-population-based feature selection methods.

## 1. Introduction

Multilabel feature selection (MLFS) involves the identification of important features that depend on a given set of labels. It is often used as an effective preprocessing step for complicated learning processes, because noisy features in relationships between multiple labels can be eliminated from subsequent training, resulting in improved multilabel classification performance [1,2]. Given an original feature set $F=\{f_{1},\ldots ,f_{\left|F\right|}\}$, MLFS identifies a feature subset S⊂F composed of n≪|F| features that are dependent on the label set $L=\{l_{1},\ldots ,l_{\left|L\right|}\}$. Conventional studies on MLFS have indicated that population-based evolutionary algorithms are promising, owing to their global search capability [3,4].

Conventional genetic algorithms that are based on a single population have suffered from premature convergence of the population, resulting in local optimal solutions [5]. Multi-population genetic algorithms (MPGAs) have recently gained significant attention as a means for circumventing the aforementioned issue. This is because they enable one sub-population to avoid premature convergence by referencing individuals or solutions from other sub-populations [6,7,8]. With regard to the feature selection problem, the communication process would improve the search capability of the sub-populations, because they can acquire hints regarding important features by referencing the best individuals from other sub-populations [9].

To the best of our knowledge, most studies have used a traditional communication process to solve the MLFS problem, even though it is intended for solving a single-label feature selection problem [4,5]. A novel communication process should be designed to maximize the benefit of using the MPGA for solving the MLFS problem. In this paper, we propose a new MPGA that specializes in solving the MLFS problem by enhancing the communication process. Specifically, an individual to be referenced is chosen from other sub-populations based on the concept of label complementarity from the viewpoint of the discriminating power corresponding to each label; then, the chosen individual is used in our improved update process. In this regard, our primary contributions are as follows:

- We proposed an MPGA that specializes in solving the MLFS problem by introducing a novel communication process and improving the update process.
- We introduced a new concept of label complementarity derived from the fact that feature subsets with a high discriminating power for different label subsets can complement each other.

## 2. Related Work

Recent MLFS methods can be broadly classified into filter-based and wrapper-based methods. The filter-based methods assess the importance of features through their own measure based on feature and label distributions. Thereafter, the top-*n* features with the highest scores are selected. Li et al. [10] proposed a granular MLFS method that attempts to select a more compact feature subset using information granules of the labels instead of the entire label set. Kashef and Nezamabadi-pour [11] proposed a Pareto dominance-based multilabel feature filter for online feature selection, which concerns the number of features being added sequentially. Gonzalez-Lopez et al. [12,13] proposed distributed models that measure the quality of each feature based on mutual information on Apache Spark. Seo et al. [14] proposed a generalized information-theoretic criterion for MLFS. They introduced entropy approximation generalized to cardinality, which was chosen by users based on the trade-off between approximation precision and computational cost. However, the classification performance of these methods is limited, because they work independently of the subsequent learning algorithm.

In contrast, wrapper-based methods evaluate the superiority of candidate feature subsets that are based on a specific learning algorithm such as a multilabel naive Bayes classifier [15]. They generally outperform the filter-based methods in terms of classification accuracy [16]. Among the wrapper-based methods, population-based evolutionary search methods are frequently used for feature selection, owing to their stochastic global search capability [17]. Lu et al. [18] proposed a new functional constriction factor to avoid premature convergence in traditional particle swarm optimization. Mafarja and Mirjalili [19] proposed binary variants of a whale optimization algorithm and applied them to the feature selection. Nakisa et al. [20] used five population-based methods in order to determine the best subset of electroencephalogram features. Dong et al. [21] improved a genetic algorithm using granular computing to select important features in high-dimensional data with a low sample size. Moreover, Lim and Kim [22] proposed an initialization method for evolutionary search-based MLFS algorithms by approximating conditional mutual information. Lee et al. [23] introduced a score function to deal with multilabel text datasets without problem transformation in a memetic search. However, these single population-based methods suffer from premature convergence of the population, resulting in limited search capability. Although methods, such as a multi-niche crowding genetic algorithm [24], can be used to mitigate premature convergence, they are still sensitive to the initialization of the population.

To resolve these issues, recent single-label feature selection studies have considered multi-population-based methods while using multiple isolated sub-populations. Ma and Xia [25] proposed a tribe competition-based genetic algorithm that attempts to ensure the diversity of solutions by allowing the sub-populations to generate feature subsets with different numbers of features. Additionally, it explores an entire search space by competitively allocating computing resources to the sub-populations. Zhang et al. [26] proposed an enhanced multi-population niche genetic algorithm. To avoid local optima, it included a process of exchanging the best individuals or solutions between the sub-populations during the search process. It also reduced the chances of similar individuals being selected as parents, based on the Hamming distance. Wang et al. [27] proposed a bacterial colony optimization method by considering a multi-dimensional population. Similar to the study that was conducted by Ma and Xia, the entire search space was divided based on the number of features selected, and the sub-populations explored different search spaces.

## 3. Label Complementary Multi-Population Genetic Algorithm for Mlfs

### 3.1. Preliminary

Table 1 summarizes the terms used for elucidating the proposed method. Conventional MPGAs for single-label feature selection entail the following processes.

Step 1: Initialization of sub-populations. Each sub-population $P_{k}$ consists of individuals whose number is a pre-defined parameter. Furthermore, each individual represents a feature subset. For example, in the genetic algorithm, each individual is represented as a binary vector called a chromosome, which comprises ones and zeros that represent selected and unselected features, respectively. In particle swarm optimization, each individual is represented as a probability vector. The components of a particle are regarded as the probabilities that the corresponding features will be selected. In most studies, the individuals are initialized randomly.

Step 2: Evaluation using a fitness function. The individuals of each sub-population can be evaluated using a fitness function. Given a feature subset represented by each individual $ind_{i}$, a learning algorithm, such as a naive Bayes classifier, is trained, and trained classifier is used to predict the label for each test pattern. Given a correct label and the predicted label, a fitness value can be computed using evaluation metrics, such as accuracy. Intuitively, a feature subset that results in better single-label prediction has better a fitness value.

Step 3: Communication among sub-populations. The sub-populations communicate with each other based on the best individuals in terms of the fitness value. In each sub-population, the worst individual (with the lowest fitness value) is replaced by the best individual of another sub-population.

Step 4: Sub-population update. The individuals generate offspring via genetic operators. First, each sub-population chooses the parents based on fitness values. For example, roulette-wheel selection employs the fitness value percentage of each individual in each subpopulation, as the probability that the individual will be chosen as a parent. Subsequently, the offspring are generated via the crossover of parents or mutation.

**Figure 1:**
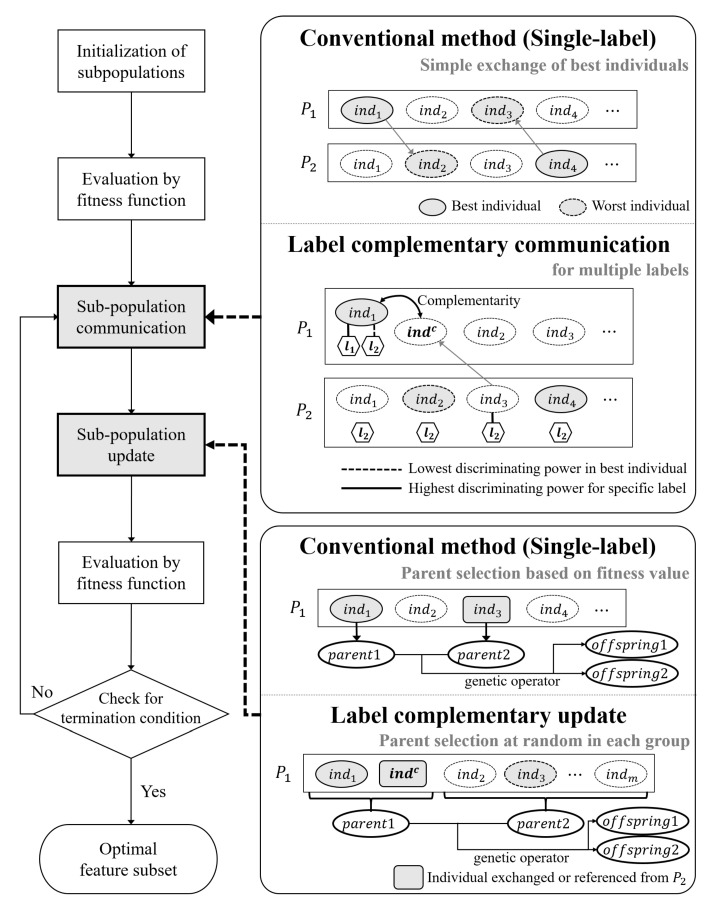
Schematic overview of proposed method.

Whenever the individuals are modified in Step 4, they are evaluated in the same manner as in Step 2. During the search process, MPGAs repeat Step 3→Step 4→Step 2 until a stopping criterion is met. In the left side of  Figure 1, the aforementioned process is presented as a flowchart.

### 3.2. Motivation and Approach

We designed a novel MPGA that specializes in solving the MLFS problem. To extend the benefits of the communication process used in conventional studies to the MLFS problem, the following issues should be considered:

- Through communication between the sub-populations, the discriminating power of multiple labels should be complemented. Additionally, the referenced individuals should be used to generate offspring that are superior to the previous generation.
- Feature subsets with high discriminating power for different label subsets can complement each other. Therefore, each sub-population should refer to an individual with the highest discriminating power for a subset of labels that are relatively difficult to discriminate, resulting in improved search capability for the MLFS.

Existing fitness-based parent selection methods may not fully use the individuals referenced from other sub-populations, because they are selected, regardless of fitness in our method. This issue can be resolved by ensuring that one of the important individuals in each sub-population is involved when generating the offspring.

 Figure 1 presents a schematic overview of the proposed MPGA for solving the MLFS problem. Particularly, we modified the communication and update process of the existing MPGA. First, with regard to sub-population communication, the conventional method communicates by exchanging the best individuals among sub-populations. Specifically, the sub-population $P_{1}$ imports the best individual $ind_{4}$ of $P_{2}$; then, the worst individual $ind_{3}$ is replaced by $ind_{4}$ of $P_{2}$. Similarly, $ind_{2}$ of $P_{2}$ is replaced by $ind_{1}$ of $P_{1}$. In the proposed label complementary communication for MLFS, the evaluation of the individuals is performed similarly to that performed in the conventional methods for single-label feature selection; however, the learning algorithm is replaced by a multilabel classification algorithm, such as a multilabel naive Bayes classifier (MLNB) [15], which uses a series of functions that predict each label. Therefore, the discriminating power corresponding to each label can be obtained by reusing the learning algorithm that was trained to evaluate the fitness values of individuals; a detailed description of this process is presented in Section 3.3. As shown in  Figure 1, the best individual $ind_{1}$ of $P_{1}$ lacks sufficient classification performance with regard to the label $l_{2}$. To complement the discriminating power with regard to $l_{2}$, $P_{1}$ refers to individual $ind_{3}$ of $P_{2}$, which best discriminates $l_{2}$.

In the sub-population updating step, the conventional method stochastically or deterministically selects the parents of $P_{1}$ via fitness-based selection. Here, the individual $ind_{3}$ that is imported from $P_{2}$ is selected and used with a high probability, because it had the highest fitness in $P_{2}$. In contrast, in the proposed label complementary updating step, the complementary individual $ind^{c}$ referenced from $P_{2}$ is chosen, regardless of fitness. Because the important individuals of $P_{1}$ are the complementary individual $ind^{c}$ and the best individual $ind_{1}$, one of them is selected as a parent. In other words, one of the important individuals is always involved in the generation of offspring. For diversity, another parent is selected from the remaining individuals at random. Finally, the selected parents generate offspring while using a genetic operator.

If a MPGA begins with a promising initial sub-populations, then a good-quality feature subset can be found by spending fewer time than that begins with a randomly-initialized sub-populations. In this study, we introduce a simple but effective initialization method. Given an original feature set $F=\{f_{1},\ldots ,f_{\left|F\right|}\}$ and the number of sub-populations *m*, the spherical *k*-means algorithm partitions *F* into *m* clusters [28]; herein, each of the clusters are composed of different features without overlapping, such that $|C_{1}\left|+\right|C_{2}\left|+\cdots +\right|C_{k}\left|+\cdots +\right|C_{m}\left|=|F\right|$. Subsequently, each sub-population $P_{k}$ is intialized based on repetitive entropy-based stochastic sampling from cluster $C_{k}$. Section 3.3 presents a detailed description of the sampling process.

### 3.3. Algorithm

Algorithm 1 represents the pseudocode of the proposed method. Each individual (chromosome) is represented by a binary string that is composed of ones and zeros, representing selected and unselected features, respectively. For simplicity, each sub-population is represented as a set of individuals, i.e., $P_{k}=\left\{ind_{1},\ldots ,ind_{|P_{k}|}\right\}$. Additionally, all of the sub-populations have the same number of individuals. In the initialization step (line 4), the individuals of each sub-population are initialized by Algorithm 2, and then evaluated to obtain their fitness values (line 6). In this study, the MLNB is used as the learning algorithm. Given the trained learning algorithm, the fitness values are computed according to the multilabel evaluation metrics detailed in Section 4.1. To evaluate the discriminating power corresponding to each label, our algorithm uses an accuracy metric used in the fitness evaluation of single-label feature selection methods. For each individual $ind_{i}$ that belongs to $P_{k}$, the label-specific accuracy vector $a_{i}=\left[a_{i1},\ldots ,a_{i|L|}\right]$ is computed by reusing the already trained learning algorithm; here, $a_{ij}$ is the accuracy corresponding to the *j*-th label predicted by $ind_{i}$. Consequently, the label-specific accuracy matrix $A_{k}\in R^{|P_{k}\left|\times |L\right|}$ is computed across all individuals of $P_{k}$ (line 7).
**Algorithm 1** Label Complementary multi-population genetic algorithm for multilabel feature selection1:**Input:**D,m;                  ▹ the multilabel dataset *D*, the number of sub-populations *m*2:**Output**: *S*;                                  ▹ the final feature subset *S*3:t←0;4:$[P_{1}\left(t\right),\ldots ,P_{m}\left(t\right)]\leftarrow$**initialization**(m)                       ▹ use **Algorithm 2**5:**for** each sub-population $P_{k}$ **do**6:    $v_{k}\left(t\right)\leftarrow$ evaluate $P_{k}\left(t\right)$ using *D*;              ▹ compute fitness values via a fitness function7:    $A_{k}\left(t\right)\leftarrow$ compute the label-specific accuracy matrix for individuals of $P_{k}\left(t\right)$;    ▹ reuse the fitness function8:**end for**9:**while** (**not** termination-condition) **do**10:    **for** each sub-population $P_{k}$ **do**11:        $ind^{c}\leftarrow$**communication**$\left(P_{k}\left(t\right),A\right)$;                       ▹ use **Algorithm 3**12:        $P_{k}\left(t+1\right)\leftarrow$**update**$\left(P_{k}\left(t\right),ind^{c}\right)$;                       ▹ use **Algorithm 4**13:        $v_{k}\left(t+1\right)\leftarrow$ evaluate $P_{k}\left(t+1\right)$ using *D*;14:        $A_{k}\left(t+1\right)\leftarrow$ compute the label-specific accuracy matrix for individuals of $P_{k}\left(t+1\right)$;15:        t←t+1;16:    **end for**17:**end while**18:S← the best feature subset so far;


**Algorithm 2** Initialization function
1:**input:***m*;                       ▹ the number of sub-populations *m*2:**output:**$P_{1},\ldots ,P_{k},\ldots ,P_{m}$;        ▹ the initial sub-populations $P_{1},\ldots ,P_{k},\ldots ,P_{m}$3:**for** each feature $f_{i}\in F$**do**                   ▹ the original feature set *F*4:    **if** $H(f_{i})=0$ **then**5:        $F\leftarrow F\backslash f_{i}$;6:    **end if**7:
**end for**
8:$[C_{1},\ldots ,C_{k},\ldots ,C_{m}]\leftarrow$ patition *F* into *m* clusters; ▹ use the spherical *k*-means algorithm9:**for**k=1 to *m* **do**10:    **for** each individual $ind_{i}\in P_{k}$ **do**11:        $ind_{i}\leftarrow$ initialize by selecting *n* features via stochastic sampling;     ▹ use Equation (1)12:    **end for**13:
**end for**



After the initialization process, the sub-populations complement each other via the proposed label complementary communication (line 11), i.e., Algorithm 3. Specifically, each sub-population identifies a complementary individual $ind^{c}$ that can complement itself from the other sub-populations. Next, our algorithm updates the sub-populations while using $ind^{c}$ via Algorithm 4. All of the sub-populations repeat these processes until the termination condition is met. We use the number of fitness function calls (FFCs) as the termination criterion, and the algorithm conducts the search until the available FFCs are exhausted. Finally, Algorithm 1 outputs the best feature subset.

Algorithm 2 represents the procedure of initialization process for each sub-population. With regard to lines 3–7, if the entropy of any feature is zero, then it is preferentially removed because it does not have any information. Each cluster $C_{k}$ of features is generated by the spherical *k*-means algorithm (line 8), and it is used to initialize each sub-population $P_{k}$ (lines 9–13). Given each feature $f_{i}^{k}\in C_{k}$, its importance score $p_{i}^{k}\in \left[0,1\right]$ is calculated as

(1) $$p_{i}^{k}=\frac{H(f_{i}^{k})}{\sum_{f\in C_{k}}H\left(f\right)}$$

where H(x)=−∑P(x)logP(x) is the entropy of a variable *x*. Finally, each individual of $P_{k}$ is initialized via stochastic sampling based on the importance scores (line 11).
**Algorithm 3** Communication function1:**input:**P,A; ▹ the sub-population *P*, the label-spcific accuracy matrix $A=\left(a_{ij}\right)\in R^{\left|P|\times |L\right|}$2:**output:**$ind^{c}$;                   ▹ the complementary individual $ind^{c}$3:b← find an index of the best individual in the *P*;        ▹ the best individual $ind_{b}$4:$L_{e}\leftarrow$ find an index set of labels with the highest error based on $a_{b}=\left[a_{b1},\ldots ,a_{b|L|}\right]$;5:**for** each individual $ind_{i}\in P^{\prime }$**do**            ▹ the other sub-populations $P^{\prime }$6:    $c_{i}\leftarrow \sum_{j\in L_{e}}a_{ij}^{\prime }$;                 ▹ the degree of complementarity *c*7:**end for**8:$ind^{c}\leftarrow$ find a individual with highest *c*;

Algorithm 3 illustrates the procedure for realizing the label complementary communication between the sub-populations for multiple labels. For simplicity, an input sub-population and the others are represented as *P* and $P^{\prime }$, respectively. With regard to lines 3–4, our algorithm finds an index set $L_{e}$ of labels for which the best individual $ind_{b}$ in *P* yields the lowest accuracies, where the size of $L_{e}$ is set to half the size of the entire label set ⌊|L|/2⌋. To find the complementary individual $ind^{c}$ from the other sub-populations $P^{\prime }$, our algorithm computes the degree of complementarity $c_{i}$ for each individual $ind_{i}$ in $P^{\prime }$, where $c_{i}$ is regarded as the discriminating power with regard to the labels in $L_{e}$. Specifically, $c_{i}$ is calculated by adding the accuracies corresponding to the labels in $L_{e}$ (line 6). In contrast with the simple communication of exchanging the best individuals, the individual $ind^{c}$ referenced from the other sub-populations can complement the discriminating power of the sub-population *P* for the entire label set *L*, which results in an improved search capability for MLFS.
**Algorithm 4** Update function1:**input:**$P\left(t\right),ind^{c}$;                      ▹ the sub-populations P(t)2:**output:**P(t+1);                     ▹ the new sub-population P(t+1)3:[o1,o2]← generate new offspring by crossover from the $ind^{c}$ and best individual of P(t);4:P(t+1)←{o1,o2};5:**while**|P(t+1)|<|P(t)|**do**                ▹ keep the number of individuals in the *P*6:    p1← select an individual at random among the $ind^{c}$ and best individual of the P(t);7:    p2← select an individual at random among remaining individuals of P(t);8:    [o1,o2]← generate new offspring by crossover from the p1 and p2;9:    P(t+1)←P(t+1)∪{o1,o2};10:**end while**11:P(t+1)← run a mutation on overlapping individuals;

Algorithm 4 represents the detailed procedure for generating new offspring. Because the complementary individual $ind^{c}$ and the best individual in P(t) are considered to be important, our algorithm generates offspring from them once (line 3–4). With regard to lines 6–7, our algorithm conducts parent selection to generate offspring. Particularly, the first parent is randomly selected between $ind^{c}$ and the best individual; consequently, the important individuals are always involved in the generation of offspring. Furthermore, to generate diverse offspring, the other parent is selected from one of the remaining individuals. As shown in line 8, the selected parent pair generates offspring via a restrictive crossover method that is frequently used to control the number of selected features in feature selection [29]. When compared to updating based on fitness-based parent selection, our algorithm can generate offspring that are superior to the previous generation by actively using the complementary individual $ind^{c}$. The generated offspring are sequentially added to P(t+1) (line 9). To maintain the number of individuals in each sub-population, the generation process is repeated until the offspring are as numerous as the number of individuals in P(t), i.e., |P(t+1)|=|P(t)|. Furthermore, as described in line 11, a restrictive mutation is conducted on overlapping individuals.

Finally, we conducted the time complexity analysis of the proposed method. The most time is spent to evaluate feature subsets, because the learning algorithm should be trained through complicated sub-procedures for multiple labels [30]. Because the numbers of training patterns and given labels are regarded as constant values during the evaluation process, the computation time required to evaluate a feature subset *S* is determined by the number of selected features |S|≤n, i.e., O(nσ), where σ represents the assumed basic time associated with the evaluation of a single feature [3]. Given the total number of individuals $N_{ind}$ and maximum number of iterations $N_{iter}$, the feature subset evaluation is conducted $N_{ind}\cdot N_{iter}$ times. Thus, the time complexity of the proposed method is $O(N_{ind}\cdot N_{iter}\cdot n\sigma )$.

### 3.4. Algorithm: Example

We implement the proposed method on the multilabel toy dataset provided in Table 2 as a representative example. In the table, each text pattern $w_{i}$ is relevant to multiple labels, where the labels are represented as one if relevant and zero otherwise. Specifically, the first pattern $w_{1}$ includes the terms “Music”, “The”, “Funny”, and “Lovely”, but not “Boring.” This pattern can be assigned to the labels “Comedy” and “Disney” simultaneously. For simplicity, we set the number of sub-populations and the number of features as two. Additionally, the number osf individuals in each sub-population was set to three. To focus on the communication process, in the initialization step, two sub-populations were initialized at random, as follows:

(2) $$\begin{array}{cc} P_{1} & =\left\{ind_{1},ind_{2},ind_{3}\right\}=\left\{10010,01100,11000\right\}\\ P_{2} & =\left\{ind_{1},ind_{2},ind_{3}\right\}=\left\{00110,10010,00101\right\} \end{array}$$

MLNB and multilabel accuracy are used to evaluate each individual. A detailed description of the evaluation metrics, including multilabel accuracy, is given in Section 4.1. Additionally, the fitness values $v_{k}$ for each sub-population $P_{k}$ are calculated as the average value obtained from 10 repeated experiments, as follows:

(3) $$\begin{array}{cc} v_{1} & =\left[0.65,0.20,0.37\right],\;A_{1}=\left[\begin{array}{ccc} 0.90 & 0.90 & 0.30\\ 0.27 & 0.33 & 0.47\\ 0.67 & 0.70 & 0.23 \end{array}\right]\\ v_{2} & =\left[0.64,0.53,0.33\right],\;A_{2}=\left[\begin{array}{ccc} 0.77 & 0.77 & 0.40\\ 1.00 & 1.00 & 0.23\\ 0.30 & 0.33 & 0.87 \end{array}\right] \end{array}$$

where the label-specific accuracy matrix $A_{k}$ for $P_{k}$ is calculated using the MLNB that was pretrained for fitness evaluation.

In the communication process for $P_{1}$, our algorithm determines the index set $L_{e}$ of labels for which the lowest accuracies are yielded by the best individual ${\mathrm{ind}}_{1}=10010$, as it has the highest fitness 0.65 in $P_{1}$. We indicate important individuals in the sub-population $P_{1}$ using bold font. In $A_{1}=\left(a_{ij}\right)$, ${\mathrm{ind}}_{1}$ has the lowest accuracy, 30% for $l_{3}$, as $\min_{l_{k}\in L}a_{1k}$ is 0.30 when k=3 because $|L_{e}|=\lfloor |L|/2\rfloor =\left\lfloor 3/2\right\rfloor =1$, $L_{e}$ = {3}. To complement $P_{1}$, our algorithm finds the complementary individual $ind^{c}$ from $P_{2}$. Based on $A_{2}$ and $L_{e}$, the degree of complementarity $c_{i}$ for each individual $ind_{i}$ of $P_{2}$ is calculated as

(4) $$\begin{array}{cc} c_{1} & =\sum_{j\in \{3\}}a_{1j}=a_{13}=0.40\\ c_{2} & =\sum_{j\in \{3\}}a_{2j}=a_{23}=0.23\\ c_{3} & =\sum_{j\in \{3\}}a_{3j}=a_{33}=0.87 \end{array}$$

Because the individual $ind_{3}$ belonging to $P_{2}$ has $c_{3}=0.87$, the complementary individual for $P_{1}$ is ${\mathrm{ind}}^{c}=00101$. Conventional methods import the best individual $ind_{1}=00110$ that belongs to $P_{2}$. Our example exhibits a low accuracy of 40% for $l_{3}$. However, our method refers to $ind_{3}=00101$ of $P_{2}$, which has the highest accuracy with regard to $l_{3}$. This indicates that our method can further complement the discriminating power of $P_{1}$ for multiple labels and increase the likelihood of avoiding local optima, resulting in improved multilabel accuracy. This process is similar for $P_{2}$.

In the update process, $P_{1}$ selects its best individual ${\mathrm{ind}}_{1}$ and ${\mathrm{ind}}^{c}$ to be the parental pair once. Next, one of ${\mathrm{ind}}_{1}$ or ${\mathrm{ind}}^{c}$ is selected as a parent, and one of $ind_{2}$ or $ind_{3}$ is selected as the other parent at random. The selected parent pair generates offspring via the genetic operators used in conventional methods. Given ${\mathrm{ind}}_{1}=10010$ and ${\mathrm{ind}}^{c}=00101$ as the parent pair, our algorithm generates offspring 00110 and 10001 via the restrictive crossover. As a result, a feature subset $\left\{f_{1},f_{5}\right\}$ represented by the offspring 10001 achieved a multilabel accuracy of 91%. This search process is repeated until the stopping criterion is met.

## 4. Experimental Results

### 4.1. Datasets and Evaluation

We conducted experiments using 17 multilabel datasets corresponding to various domains; these datasets can be obtained from http://mulan.sourceforge.net/datasets-mlc.html [31]. Specifically, the Emotions dataset [32] consists of 8 rhythmic features and 64 timbre features. The Enron dataset [33] was sampled from a large email message set, the Enron corpus. The Genbase and Yeast datasets [34,35] contain information regarding the functions of biological proteins and genes. The Medical dataset [36] is a subset of a large corpus that is associated with suicide letters in clinical free text. The Scene dataset [37] has indexing information on still images containing multiple objects. The remaining 11 datasets were obtained from the Yahoo dataset collection [38], composed of more than 10,000 features. Table 3 indicates standard statistics for the 17 datasets used in our experiments. It includes the number of patterns |W|, number of features |F|, types of features, and number of labels |L|. If the feature type was numeric, we discretized the features while using label-attribute interdependence maximization, which is a discretization method that is specialized for multilabel data [39]. The label cardinality Card. represents the average number of labels in each pattern, and label density Den. is the label cardinality for the total number of labels. Further, Distinct. indicates the number of unique label subsets in *L*, and Domain represents the applications that are related to each dataset.

We compared the proposed method with three state-of-the-art multi-population-based methods that have exhibited promising performance for solving the feature selection problem: TCbGA [25], EMPNGA [26], and BCO-MDP [27]. We set the parameters for each method to the values used in the corresponding original study. For fairness, we set the maximum number of allowable FFCs and selected features to 300 and 50, respectively. The total population size was set to 50. The MLNB and a holdout cross-validation method were used in order to evaluate the quality of the feature subsets obtained by each method. Furthermore, 80% and 20% of each dataset were used as the training and test sets, respectively. We repeated each experiment 10 times and used the average value of the results. In the proposed method, we set the number of sub-populations to five; thus, each sub-population size was 10.

We used four evaluation metrics to evaluate the quality of the feature subsets: Hamming loss, one-error, multilabel accuracy, and subset accuracy [40,41,42]. Let $T=\{\left(w_{i},\lambda_{i}\right)|1\leq i\leq |T|\}$ be a given test set, where $\lambda_{i}\subseteq L$ is a correct label subset that is associated with a pattern $w_{i}$. Given a test pattern $w_{i}$ and a multilabel classifier, such as MLNB, estimate a predicted label set $Y_{i}\subseteq L$. Specifically, a series of functions $\left\{g_{1},g_{2},\ldots ,g_{\left|L\right|}\right\}$ is induced from the training patterns. Next, each function $g_{k}$ determines the class membership of $l_{k}$ with respect to each pattern, i.e., $Y_{i}=\{l_{k}|g_{k}\left(w_{i}\right)>\theta ,1\leq k\leq \left|L|\right\}$, where θ is a predetermined threshold, such as 0.5. The four metrics can be computed given λ and *Y* according to the test patterns. The Hamming loss is defined as

(5) $$hloss\left(T\right)=\frac{1}{\left|T\right|}\sum_{i=1}^{\left|T\right|}\frac{1}{\left|L\right|}\left|\lambda_{i}△Y_{i}\right|$$

where △ denotes the symmetric difference between two sets. Furthermore, one-error is defined as

(6) $$onerr\left(T\right)=\frac{1}{\left|T\right|}\sum_{i=1}^{\left|T\right|}\;[\arg\max_{l_{k}\in L}g_{k}\left(w_{i}\right)\notin \lambda_{i}]$$

where [·] returns one if the proposition stated in the brackets is true and zero otherwise. Multilabel accuracy is defined as

(7) $$mlacc\left(T\right)=\frac{1}{\left|T\right|}\sum_{i=1}^{\left|T\right|}\frac{|\lambda_{i}\cap Y_{i}|}{|\lambda_{i}\cup Y_{i}|}$$

It computes the Jaccard coefficient between two sets. Finally, subset accuracy is defined as

(8) $$setacc\left(T\right)=\frac{1}{\left|T\right|}\sum_{i=1}^{\left|T\right|}\;[\lambda_{i}=Y_{i}]$$

It determines whether two sets are exactly identical. A superior feature subset will exhibit higher values of the multilabel and subset accuracies and lower values of the Hamming loss and one-error metrics.

We conducted additional statistical tests in order to verify the statistical significance of our results. First, we conducted a paired *t*-test [43] at 95% significance level to compare the proposed method with each of other MLFS methods on each of datasets; because there are three comparison algorithms, the paired *t*-test is performed three times. Here, three null hypotheses (i.e., two methods have equal performance) can either be rejected or accepted. We also performed the Bonferroni–Dunn test in order to compare the average ranks of the proposed and other methods [44]. If the difference between the average rank of one comparison method and that of the proposed method is within the critical difference (CD), its performance is considered to be similar to that of the proposed method. In our experiments, we set the significance level α to 0.05, and, thus, the CD can be computed as 1.0601 [45].

### 4.2. Comparison Results

Table 4, Table 5, Table 6 and Table 7 present the experimental results of the proposed method and compare them with those of the other methods on 17 multilabel datasets. The resulting values are represented by their average performances with the corresponding standard deviations; herein, a better average value is indicated by bold font on each dataset. In addition, for each dataset, the paired *t*-test was conducted at the 95% significance level. As shown in Table 4, Table 5, Table 6 and Table 7, ▼(Δ) indicates that the corresponding method is significantly worse(better) than the proposed method based on the paired *t*-test. Table 4 shows that the proposed method is statistically superior or similar than TCbGA on 88% of the datasets and than EMPNGA and BCO-MDP on all datasets in terms of the Hamming loss. Table 5 shows that the proposed method is statistically superior or similar than other methods on 94% of the datasets in terms of the one-error. Particularly, Table 6 and Table 7 show that the proposed method is statistically superior or similar than other methods on all datasets in terms of the multilabel accuracy and the subset accuracy.

**Figure 2:**
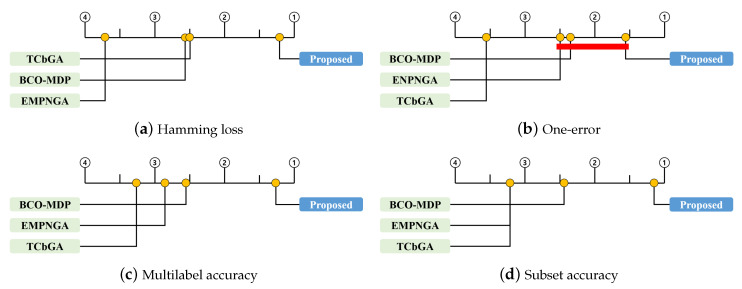
Bonferroni-Dunn test results of four comparison methods with four evaluation measures.

Figure 2 illustrates the CD diagrams, showing the relative performance of the four methods. Here, the horizontal axis represents the average rank of each method, where the higher ranks are placed on the right side of each subfigure. In addition, the methods within the same CD as that of the proposed method are connected by a bold red line, which means that the difference among them is not significant. Figure 2b indicates that the proposed method significantly outperformed the TCbGA and BCO-MDP in terms of the one-error. The results for the one-error indicate that the simple communication of exchanging the best individuals in the EMPNGA can also yield good results, because the one-error is evaluated based only on the label predicted with the highest probability. In contrast, Figure 2a,c,d indicates that the proposed method significantly outperformed all other methods in terms of the Hamming loss, multilabel accuracy, and subset accuracy. The three metrics are evaluated based on the predicted label subsets; thus, the proposed method, which employs label complementary communication, can outperform the existing methods.

### 4.3. Analysis

**Figure 3:**
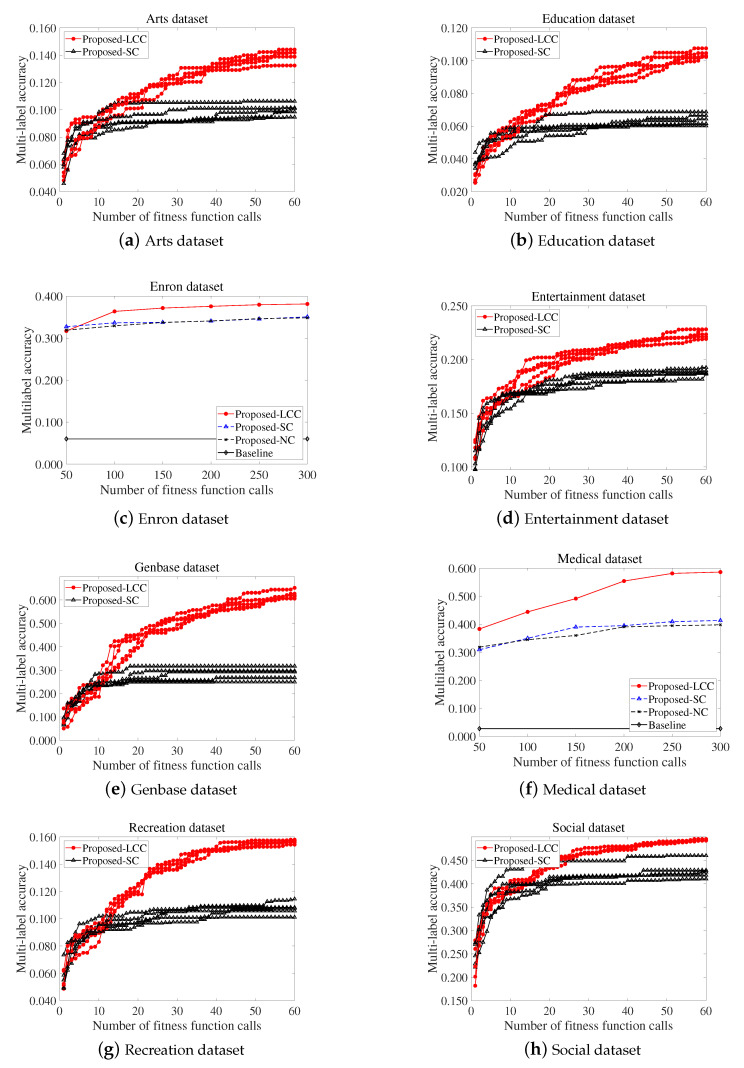
Multilabel accuracy(↑) for the best individual in each sub-population, obtained using three methods.

We conducted an in-depth analysis to determine whether the proposed communication process is effective for solving the MLFS problem via additional experiments on eight datasets using the MLNB. To validate the effectiveness of label complementary communication in the proposed method, we designed Proposed-SC, which is equivalent to the proposed method, except that it does not include the proposed communication process, i.e., Algorithm 3. Specifically, the Proposed-SC uses the simple communication method of exchanging the best individuals and roulette wheel selection as the fitness-based parent selection method. For improved readability, we named the proposed method described in Section 3 as the Proposed-LCC. In addition, we designed Proposed-NC, which is equivalent to Proposed-SC, except that it does not conduct any communication process. Figure 3 shows the search capability of each sub-population during the search process. The vertical axis indicates the multilabel accuracy for the best individual in each method; herein, the baseline indicates the multilabel accuracy obtained by random prediction from 10 repetitions and it is regarded as the baseline performance. As stated in Section 4, the numbers of maximum FFCs and the total number of individuals are 300 and 50, respectively. Therefore, the sub-populations communicate with each other every 50 FFCs. Additionally, the number of sub-populations is five.

As shown in Figure 3, the Proposed-LCC exhibited a better search capability than Proposed-SC and Proposed-NC on eight multilabel datasets. We note that, in MLFS, Proposed-SC and Proposed-NC exhibited a similar level of search capability in MLFS, and it even revealed worse search capability than a method without communication on the Education datasets. It implies that the simple communication method of exchanging the best individuals failed to deal with the multiple labels. In contrast, Proposed-LCC conducted effective MLFS searches. Particularly, in Figure 3c, the initial sub-populations of Proposed-LCC revealed relatively low multilabel accuracy (50 FFCs). This is because each of sub-populations consists of different features by our initialization method and, thus, may not be related to entire label set. During search process, Proposed-LCC exhibited an effective improvement in multilabel accuracy. This indicates that the proposed label complementary communication method can improve the search capability of the sub-populations by referencing individuals from other sub-populations based on the discriminating power of subsets with regard to labels that are difficult to classify.

**Figure 4:**
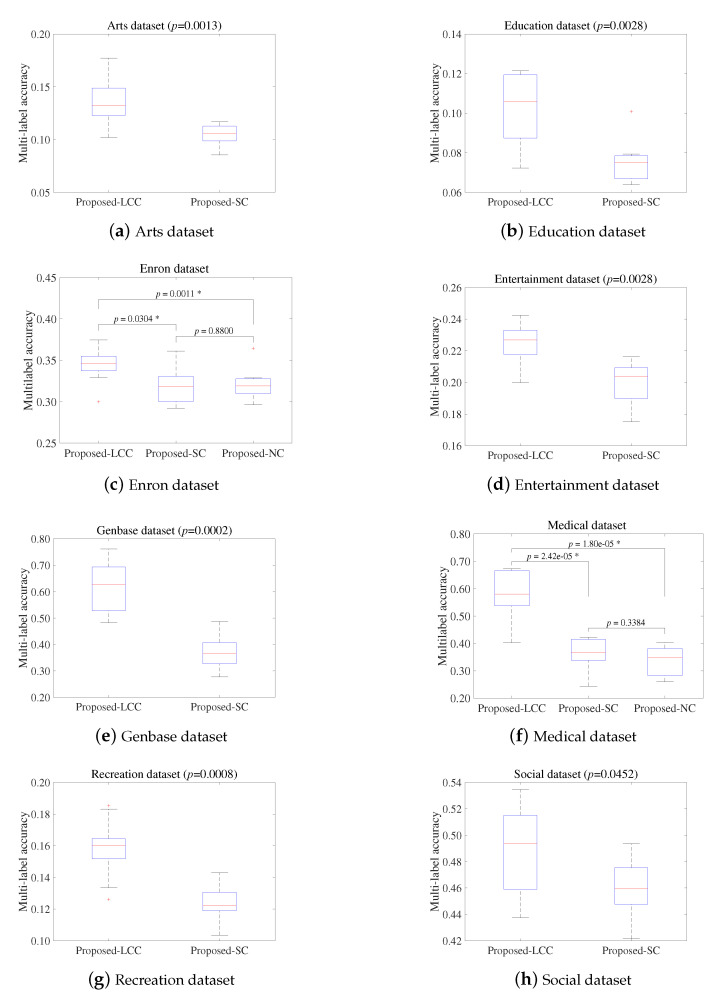
Pairwise comparison results of paired *t*-test at 95% significance level in terms of multilabel accuracy(↑).

We also conducted the paired *t*-test at 95% significance level in order to determine whether the three methods were statistically different. For fairness, Proposed-SC and Proposed-NC also obtained results from 10 repetitions on the eight datasets, respectively. Figure 4 presents the pairwise comparison results on each of datasets in terms of the multilabel accuracy; the *p*-values for each of tests are shown in each subfigure and the asterisk indicates that corresponding hypothesis was rejected. As shown in Figure 4, the Proposed-LCC significantly outperformed Proposed-SC on seven datasets, except for the Recreation dataset and outperformed Proposed-NC on all datasets. On the other hand, Proposed-SC and Proposed-NC have equal performance on all datasets. As a result, the additional experiment and statistical test verify that the proposed label complementary communication successfully improves the search capability of the sub-populations with regard to MLFS.

## 5. Conclusions

In this paper, we proposed a novel MPGA, with label complementary communication, which specializes in solving the MLFS problem. It is aimed at improving the search capability of sub-populations through a communication process that employs the complementary discriminating powers of sub-populations with regard to multiple labels. Our experimental results and statistical tests verified that the proposed method significantly outperformed three state-of-the-art multi-population-based feature selection methods on 17 multilabel datasets.

Future studies can be conducted to overcome the limitation of the proposed method: we have simply set the number of labels to be complemented to half the total number of labels. As the search progresses, this value can be adjusted according to the improvement in the discriminating power for each label. For example, the proposed label complementary communication may only be conducted for labels for which the discrimination performance is not better than that in the previous generation.

## Figures and Tables

**Table 1:** Notation used for describing/elucidating the proposed method.

Terms	Meanings
*D*	A multilabel dataset
*L*	A label set in *D*, $L=\{l_{1},\ldots ,l_{\left|L\right|}\}$
*F*	A feature set in *D*, $F=\{f_{1},\ldots ,f_{\left|F\right|}\}$
*S*	A final feature subset, |S|≤n
*t*	Number of generations
*m*	Number of sub-populations
*n*	Maximum number of selected features
$ind_{i}$	An *i*-th individual
$P_{k}$	A *k*-th sub-population, $P_{k}=\left\{ind_{1},\ldots ,ind_{|P_{k}|}\right\}$
$v_{k}$	Fitness values for the individuals of the $P_{k}$
$A_{k}$	Label-specific accuracy matrix for individuals of $P_{k}$, $A_{k}=\left(a_{ij}\right)\in R^{|P_{k}\left|\times |L\right|}$
$ind^{c}$	A complementary individual
$c_{i}$	A degree of complementarity for $ind_{i}$

**Table 2:** Multilabel toy dataset.

	Features						Labels		
Pattern	$f_{1}$	$f_{2}$	$f_{3}$	$f_{4}$	$f_{5}$		$l_{1}$	$l_{2}$	$l_{3}$
	Boring	Music	The	Funny	Lovely		Comedy	Documentary	Disney
$w_{1}$	0	1	1	1	1		1	0	1
$w_{2}$	1	0	1	0	1		0	1	1
$w_{3}$	1	1	1	0	1		0	1	1
$w_{4}$	0	1	0	1	0		1	0	0
$w_{5}$	0	0	1	1	0		1	0	0
$w_{6}$	1	0	0	0	0		0	1	0
$w_{7}$	0	0	1	1	1		1	0	1

**Table 3:** Standard statistics of multilabel datasets.

Dataset	|W|	|F|	Type	|L|	Card.	Den.	Distinct.	Domain
Arts	7484	23,146	Numeric	26	1.654	0.064	599	Text
Business	11,214	21,924	Numeric	30	1.599	0.053	233	Text
Computers	12,444	34,096	Numeric	33	1.507	0.046	428	Text
Education	12,030	27,534	Numeric	33	1.463	0.044	511	Text
Emotions	593	72	Numeric	6	1.869	0.311	27	Music
Enron	1702	1001	Nominal	53	3.378	0.064	753	Text
Entertainment	12,730	32,001	Numeric	21	1.414	0.067	337	Text
Genbase	662	1185	Nominal	27	1.252	0.046	32	Biology
Health	9205	30,605	Numeric	32	1.644	0.051	335	Text
Medical	978	1449	Nominal	45	1.245	0.028	94	Text
Recreation	12,828	30,324	Numeric	22	1.429	0.065	530	Text
Reference	8027	39,679	Numeric	33	1.174	0.036	275	Text
Scene	2407	294	Numeric	6	1.074	0.179	15	Image
Science	6428	37,187	Numeric	40	1.450	0.036	457	Text
Social	12,111	52,350	Numeric	29	1.279	0.033	361	Text
Society	14,512	31,802	Numeric	27	1.670	0.062	1054	Text
Yeast	2417	103	Numeric	14	4.237	0.303	198	Biology

**Table 4:** Comparison results of four methods in terms of Hamming loss(↓) (▼/Δ indicates that the corresponding method is significantly worse/better than proposed method based on paired *t*-test at 95% significance level).

Dataset	Proposed	TCbGA	EMPNGA	BCO-MDP
Arts	**0.0629 ± 0.001**	0.0635 ± 0.001	0.0642 ± 0.001 ▼	0.0638 ± 0.001 ▼
Business	0.0297 ± 0.001	**0.0289 ± 0.001** Δ	0.0297 ± 0.001	0.0293 ± 0.001
Computers	**0.0428 ± 0.001**	0.0432 ± 0.001	0.0435 ± 0.001	0.0435 ± 0.001
Education	**0.0443 ± 0.001**	0.0444 ± 0.000	0.0449 ± 0.001	0.0447 ± 0.001
Emotions	**0.2336 ± 0.022**	0.2370 ± 0.013	0.2376 ± 0.023	0.2366 ± 0.032
Enron	0.0663 ± 0.006	**0.0628 ± 0.004**	0.0892 ± 0.008 ▼	0.0840 ± 0.007 ▼
Entertainment	**0.0641 ± 0.001**	0.0650 ± 0.002	0.0646 ± 0.002	0.0650 ± 0.002
Genbase	**0.0074 ± 0.003**	0.0338 ± 0.006 ▼	0.0315 ± 0.004 ▼	0.0277 ± 0.006 ▼
Health	**0.0465 ± 0.003**	0.0498 ± 0.001 ▼	0.0490 ± 0.001 ▼	0.0489 ± 0.002
Medical	**0.0138 ± 0.002**	0.0206 ± 0.003 ▼	0.0186 ± 0.001 ▼	0.0181 ± 0.003 ▼
Recreation	**0.0626 ± 0.001**	0.0638 ± 0.001 ▼	0.0638 ± 0.001 ▼	0.0641 ± 0.002 ▼
Reference	**0.0342 ± 0.002**	0.0359 ± 0.000 ▼	0.0358 ± 0.001	0.0358 ± 0.001 ▼
Scene	**0.1341 ± 0.007**	0.1372 ± 0.007	0.1416 ± 0.006 ▼	0.1396 ± 0.012
Science	0.0367 ± 0.001	**0.0362 ± 0.001** Δ	0.0376 ± 0.001	0.0368 ± 0.001
Social	**0.0297 ± 0.002**	0.0323 ± 0.001 ▼	0.0309 ± 0.001 ▼	0.0315 ± 0.002 ▼
Society	**0.0586 ± 0.001**	0.0598 ± 0.001 ▼	0.0595 ± 0.001 ▼	0.0590 ± 0.001
Yeast	**0.2208 ± 0.009**	0.2233 ± 0.007	0.2253 ± 0.005	0.2241 ± 0.006
Avg. Rank	**1.24**	2.71	3.35	2.71

**Table 5:** Comparison results of four methods in terms of one-error(↓) (▼/Δ indicates that the corresponding method is significantly worse/better than the proposed method based on paired *t*-test at 95% significance level).

Dataset	Proposed	TCbGA	EMPNGA	BCO-MDP
Arts	**0.7354 ± 0.140**	0.7717 ± 0.120 ▼	0.7684 ± 0.122 ▼	0.7640 ± 0.126 ▼
Business	**0.3930 ± 0.417**	0.3935 ± 0.418	0.3933 ± 0.418	0.3935 ± 0.418
Computers	**0.4530 ± 0.011**	0.4616 ± 0.008 ▼	0.4566 ± 0.009	0.4626 ± 0.008 ▼
Education	**0.6520 ± 0.020**	0.6756 ± 0.011 ▼	0.6777 ± 0.011 ▼	0.6776 ± 0.014 ▼
Emotions	0.2992 ± 0.029	0.3085 ± 0.054	**0.2915 ± 0.060**	0.2992 ± 0.068
Enron	**0.5797 ± 0.327**	0.5982 ± 0.318	0.6074 ± 0.317 ▼	0.5976 ± 0.316 ▼
Entertainment	**0.6085 ± 0.023**	0.6710 ± 0.023 ▼	0.6339 ± 0.014 ▼	0.6483 ± 0.023 ▼
Genbase	**0.7197 ± 0.441**	0.8652 ± 0.207	0.8235 ± 0.272	0.8045 ± 0.303
Health	**0.7659 ± 0.299**	0.7935 ± 0.266 ▼	0.7900 ± 0.270 ▼	0.7885 ± 0.272 ▼
Medical	**0.7713 ± 0.293**	0.8395 ± 0.206	0.8138 ± 0.236	0.8287 ± 0.216
Recreation	**0.7062 ± 0.035**	0.7531 ± 0.010 ▼	0.7533 ± 0.014 ▼	0.7482 ± 0.021 ▼
Reference	0.7130 ± 0.247	0.7171 ± 0.243	**0.7126 ± 0.247**	0.7164 ± 0.244
Scene	0.3168 ± 0.029	0.2927 ± 0.027 Δ	**0.2844 ± 0.026** Δ	0.2871 ± 0.023 Δ
Science	**0.7097 ± 0.019**	0.7342 ± 0.019 ▼	0.7265 ± 0.018 ▼	0.7445 ± 0.013 ▼
Social	**0.4872 ± 0.183**	0.5637 ± 0.161 ▼	0.5441 ± 0.164 ▼	0.5677 ± 0.156 ▼
Society	0.4880 ± 0.019	0.4963 ± 0.013	**0.4859 ± 0.019**	0.4901 ± 0.014
Yeast	**0.2369 ± 0.023**	0.2431 ± 0.019	0.2652 ± 0.020 ▼	0.2513 ± 0.019 ▼
Avg. Rank	**1.35**	3.41	2.41	2.76

**Table 6:** Comparison results of four methods in terms of multilabel accuracy(↑) (▼/Δ indicates that the corresponding method is significantly worse/better than proposed method based on paired *t*-test at the 95% significance level).

Dataset	Proposed	TCbGA	EMPNGA	BCO-MDP
Arts	**0.0924 ± 0.021**	0.0330 ± 0.007 ▼	0.0464 ± 0.009 ▼	0.0518 ± 0.016 ▼
Business	0.6772 ± 0.009	**0.6784 ± 0.008**	0.6767 ± 0.011	0.6760 ± 0.010
Computers	0.4155 ± 0.008	0.4148 ± 0.007	**0.4159 ± 0.010**	0.4147 ± 0.010
Education	**0.0748 ± 0.026**	0.0291 ± 0.007 ▼	0.0367 ± 0.015 ▼	0.0410 ± 0.022 ▼
Emotions	0.5323 ± 0.036	0.5267 ± 0.035	0.5202 ± 0.031	**0.5329 ± 0.031**
Enron	**0.3445 ± 0.021**	0.3315 ± 0.019	0.3173 ± 0.019 ▼	0.3389 ± 0.034
Entertainment	**0.1904 ± 0.051**	0.0586 ± 0.022 ▼	0.1116 ± 0.016 ▼	0.1218 ± 0.046 ▼
Genbase	**0.8907 ± 0.058**	0.3789 ± 0.130 ▼	0.4238 ± 0.088 ▼	0.5471 ± 0.157 ▼
Health	**0.4277 ± 0.027**	0.4074 ± 0.016	0.4120 ± 0.019	0.4026 ± 0.015 ▼
Medical	**0.5772 ± 0.089**	0.3545 ± 0.084 ▼	0.3628 ± 0.055 ▼	0.4498 ± 0.117 ▼
Recreation	**0.1001 ± 0.026**	0.0477 ± 0.012 ▼	0.0574 ± 0.007 ▼	0.0573 ± 0.017 ▼
Reference	0.4048 ± 0.015	0.3568 ± 0.125	**0.4066 ± 0.012**	0.4005 ± 0.011
Scene	**0.5730 ± 0.038**	0.5663 ± 0.021	0.5705 ± 0.016	0.5712 ± 0.034
Science	**0.0744 ± 0.041**	0.0256 ± 0.008 ▼	0.0360 ± 0.011 ▼	0.0385 ± 0.011 ▼
Social	**0.4935 ± 0.047**	0.0720 ± 0.027 ▼	0.1907 ± 0.168 ▼	0.1187 ± 0.033 ▼
Society	0.2423 ± 0.135	0.1617 ± 0.162	0.2586 ± 0.165	**0.2873 ± 0.126**
Yeast	**0.4468 ± 0.012**	0.4435 ± 0.012	0.4448 ± 0.012	0.4418 ± 0.013
Avg. Rank	**1.35**	3.53	2.59	2.53

**Table 7:** Comparison results of four methods in terms of subset accuracy(↑) (▼/Δ indicates that the corresponding method is significantly worse/better than proposed method based on paired *t*-test at the 95% significance level).

Dataset	Proposed	TCbGA	EMPNGA	BCO-MDP
Arts	**0.0666 ± 0.015**	0.0287 ± 0.009 ▼	0.0422 ± 0.010 ▼	0.0438 ± 0.016 ▼
Business	0.5326 ± 0.011	0.5326 ± 0.012	0.5322 ± 0.012	**0.5334 ± 0.013**
Computers	**0.3386 ± 0.012**	0.3365 ± 0.007	0.3379 ± 0.009	0.3318 ± 0.007 ▼
Education	**0.0599 ± 0.019**	0.0162 ± 0.006 ▼	0.0327 ± 0.013 ▼	0.0317 ± 0.010 ▼
Emotions	0.2534 ± 0.039	**0.2593 ± 0.043**	0.2508 ± 0.041	0.2525 ± 0.055
Enron	0.1076 ± 0.020	**0.1168 ± 0.020**	0.0418 ± 0.028 ▼	0.0947 ± 0.034
Entertainment	**0.1709 ± 0.051**	0.0791 ± 0.025 ▼	0.0903 ± 0.023 ▼	0.0862 ± 0.033 ▼
Genbase	**0.8485 ± 0.041**	0.2576 ± 0.092 ▼	0.4288 ± 0.070 ▼	0.5098 ± 0.123 ▼
Health	**0.3386 ± 0.028**	0.3160 ± 0.014 ▼	0.3293 ± 0.017	0.3129 ± 0.017 ▼
Medical	**0.4636 ± 0.071**	0.2600 ± 0.047 ▼	0.3472 ± 0.049 ▼	0.3138 ± 0.096 ▼
Recreation	**0.0829 ± 0.021**	0.0393 ± 0.016 ▼	0.0475 ± 0.011 ▼	0.0478 ± 0.021 ▼
Reference	0.3579 ± 0.014	0.3532 ± 0.009	**0.3654 ± 0.013**	0.3265 ± 0.112
Scene	0.4341 ± 0.025	0.4168 ± 0.033	0.3819 ± 0.027 ▼	**0.4472 ± 0.028**
Science	**0.0602 ± 0.030**	0.0258 ± 0.003 ▼	0.0351 ± 0.011 ▼	0.0311 ± 0.008 ▼
Social	**0.4185 ± 0.051**	0.0667 ± 0.036 ▼	0.2850 ± 0.183 ▼	0.0981 ± 0.041 ▼
Society	0.2222 ± 0.060	0.1187 ± 0.132	0.1926 ± 0.125	**0.2257 ± 0.116**
Yeast	0.1029 ± 0.014	0.0969 ± 0.014	**0.1085 ± 0.006**	0.0988 ± 0.016
Avg. Rank	**1.41**	3.29	2.59	2.65

## References

[1] Gu S., Cheng R., Jin Y. (2018). Feature selection for high-dimensional classification using a competitive swarm optimizer. Soft Comput..

[2] Zawbaa H.M., Emary E., Grosan C., Snasel V. (2018). Large-dimensionality small-instance set feature selection: A hybrid bio-inspired heuristic approach. Swarm Evol. Comput..

[3] Lee J., Kim D.W. (2015). Memetic feature selection algorithm for multi-label classification. Inf. Sci..

[4] Pereira R.B., Plastino A., Zadrozny B., Merschmann L.H. (2018). Categorizing feature selection methods for multi-label classification. Artif. Intell. Rev..

[5] Ma H., Shen S., Yu M., Yang Z., Fei M., Zhou H. (2019). Multi-population techniques in nature inspired optimization algorithms: A comprehensive survey. Swarm Evol. Comput..

[6] Li C., Nguyen T.T., Yang M., Yang S., Zeng S. (2015). Multi-population methods in unconstrained continuous dynamic environments: The challenges. Inf. Sci..

[7] Nseef S.K., Abdullah S., Turky A., Kendall G. (2016). An adaptive multi-population artificial bee colony algorithm for dynamic optimisation problems. Knowl.-Based Syst..

[8] Li J.Y., Zhao Y.D., Li J.H., Liu X.J. (2015). Artificial bee colony optimizer with bee-to-bee communication and multipopulation coevolution for multilevel threshold image segmentation. Math. Probl. Eng..

[9] Qiu C. (2019). A novel multi-swarm particle swarm optimization for feature selection. Genet. Program. Evol. Mach..

[10] Li F., Miao D., Pedrycz W. (2017). Granular multi-label feature selection based on mutual information. Pattern Recognit..

[11] Kashef S., Nezamabadi-pour H. (2019). A label-specific multi-label feature selection algorithm based on the Pareto dominance concept. Pattern Recognit..

[12] González-López J., Ventura S., Cano A. (2019). Distributed selection of continuous features in multilabel classification using mutual information. IEEE Trans. Neural Netw. Learn. Syst..

[13] Gonzalez-Lopez J., Ventura S., Cano A. (2020). Distributed multi-label feature selection using individual mutual information measures. Knowl.-Based Syst..

[14] Seo W., Kim D.W., Lee J. (2019). Generalized Information-Theoretic Criterion for Multi-Label Feature Selection. IEEE Access.

[15] Zhang M.L., Peña J.M., Robles V. (2009). Feature selection for multi-label naive Bayes classification. Inf. Sci..

[16] Cai J., Luo J., Wang S., Yang S. (2018). Feature selection in machine learning: A new perspective. Neurocomputing.

[17] Xue B., Zhang M., Browne W.N., Yao X. (2016). A survey on evolutionary computation approaches to feature selection. IEEE Trans. Evol. Comput..

[18] Lu Y., Liang M., Ye Z., Cao L. (2015). Improved particle swarm optimization algorithm and its application in text feature selection. Appl. Soft Comput..

[19] Mafarja M., Mirjalili S. (2018). Whale optimization approaches for wrapper feature selection. Appl. Soft Comput..

[20] Nakisa B., Rastgoo M.N., Tjondronegoro D., Chandran V. (2018). Evolutionary computation algorithms for feature selection of EEG-based emotion recognition using mobile sensors. Expert Syst. Appl..

[21] Dong H., Li T., Ding R., Sun J. (2018). A novel hybrid genetic algorithm with granular information for feature selection and optimization. Appl. Soft Comput..

[22] Lim H., Kim D.W. (2020). MFC: Initialization method for multi-label feature selection based on conditional mutual information. Neurocomputing.

[23] Lee J., Yu I., Park J., Kim D.W. (2019). Memetic feature selection for multilabel text categorization using label frequency difference. Inf. Sci..

[24] Breaban M., Luchian H. (2011). A unifying criterion for unsupervised clustering and feature selection. Pattern Recognit..

[25] Ma B., Xia Y. (2017). A tribe competition-based genetic algorithm for feature selection in pattern classification. Appl. Soft Comput..

[26] Zhang W., He H., Zhang S. (2019). A novel multi-stage hybrid model with enhanced multi-population niche genetic algorithm: An application in credit scoring. Expert Syst. Appl..

[27] Wang H., Tan L., Niu B. (2019). Feature selection for classification of microarray gene expression cancers using Bacterial Colony Optimization with multi-dimensional population. Swarm Evol. Comput..

[28] Dhillon I.S., Modha D.S. (2001). Concept decompositions for large sparse text data using clustering. Mach. Learn..

[29] Zhu Z., Ong Y.S., Dash M. (2007). Wrapper–filter feature selection algorithm using a memetic framework. IEEE Trans. Syst. Man Cybern. B Cybern..

[30] Lee J., Park J., Kim H.C., Kim D.W. (2019). Competitive Particle Swarm Optimization for Multi-Category Text Feature Selection. Entropy.

[31] Tsoumakas G., Spyromitros-Xioufis E., Vilcek J., Vlahavas I. (2011). Mulan: A java library for multi-label learning. J. Mach. Learn. Res..

[32] Trohidis K., Tsoumakas G., Kalliris G., Vlahavas I.P. (2008). Multi-Label Classification of Music into Emotions. Proceedings of the 9th International Conference of Music Information Retrieval (ISMIR).

[33] Klimt B., Yang Y. (2004). The Enron Corpus: A New Dataset for Email Classification Research.

[34] Diplaris S., Tsoumakas G., Mitkas P.A., Vlahavas I. (2005). Protein Classification with Multiple Algorithms.

[35] Elisseeff A., Weston J. A kernel method for multi-labelled classification. Proceedings of the International Conference on Neural Information Processing Systems: Natural and Synthetic.

[36] Pestian J., Brew C., Matykiewicz P., Hovermale D.J., Johnson N., Cohen K.B., Duch W. (2007). A shared task involving multi-label classification of clinical free text. Biological, Translational, and Clinical Language Processing.

[37] Boutell M.R., Luo J., Shen X., Brown C.M. (2004). Learning multi-label scene classification. Pattern Recognit..

[38] Ueda N., Saito K. Parametric mixture models for multi-labeled text. Proceedings of the International Conference on Neural Information Processing Systems.

[39] Cano A., Luna J.M., Gibaja E.L., Ventura S. (2016). LAIM discretization for multi-label data. Inf. Sci..

[40] Madjarov G., Kocev D., Gjorgjevikj D., Džeroski S. (2012). An extensive experimental comparison of methods for multi-label learning. Pattern Recognit..

[41] Pereira R.B., Plastino A., Zadrozny B., Merschmann L.H. (2018). Correlation analysis of performance measures for multi-label classification. Inf. Process. Manag..

[42] Zhang M.L., Zhou Z.H. (2014). A review on multi-label learning algorithms. IEEE Trans. Knowl. Data Eng..

[43] McDonald J.H. (2009). Handbook of Biological Statistics.

[44] Dunn O.J. (1961). Multiple comparisons among means. J. Am. Stat. Assoc..

[45] Demšar J. (2006). Statistical comparisons of classifiers over multiple data sets. J. Mach. Learn. Res..

